# The Dosimetric Analysis of Duodenal and Intestinal Toxicity After a Curative Dose Re-irradiation Using the Intensity-Modulated Radiotherapy for Abdominopelvic Lymph Node Lesions

**DOI:** 10.7759/cureus.50920

**Published:** 2023-12-21

**Authors:** Asami Yoshida, Satoaki Nakamura, Ryoong-Jin Oh, Hiroya Shiomi, Hideya Yamazaki, Ken Yoshida, Noboru Tanigawa

**Affiliations:** 1 Radiation Oncology, Kansai Medical University, Hirakata, JPN; 2 Radiation Oncology, Miyakojima Image Guided Radiation Therapy (IGRT) Clinic, Osaka, JPN; 3 Radiology, Kyoto Prefectural University of Medicine, Kyoto, JPN; 4 Radiology, Kansai Medical University, Hirakata, JPN

**Keywords:** intensity-modulated radiation therapy, pelvic, abdominal, dose-volume histogram, re-irradiation

## Abstract

Introduction: This study aimed to examine the influence of dosimetric factors on gastrointestinal toxicity after radical re-irradiation for lymph node recurrence in the abdominopelvic region using a composite plan.

Methods: Between January 2008 and March 2017, 33 patients underwent radical re-irradiation for lymph node recurrence in the abdominopelvic region with a complete overlap with previous radiation therapy (RT) with the median prescription dose of the second RT of 71.7 Gy_10_. Re-irradiation planning protocol for target volume and organs at risk (OARs) (duodenum, small and large intestines) was decided as follows: more than equal to 97% of the prescription dose was administered to the D95 (percentage of the minimum dose that covered 95% of the target volume) of planning target volume (PTV); minimal dose to the maximally irradiated doses delivered to 1cc [D1 cc] and 5cc [D5 cc] of OARs was set below 70 Gy_3_ and 50 Gy_3_, respectively; and D1 cc and D5 cc in the cumulative plans to OARs were 120 Gy_3_ and 100 Gy_3_. Kaplan-Meier analyses were performed to evaluate overall survival (OS) and univariate log-rank and multivariate Cox proportional hazards model analyses were performed to explore predictive factors. Using dose summation of the first and re-irradiation plans, we conducted a dosimetric analysis for grade ≥ 3 toxicities of the duodenum and intestine.

Results: With a median follow-up of 18 months, the two-year OS rate was 45.5%. The number of RT fields (localized or multiple) was a significant predisposing factor for OS rate with a hazard ratio of 0.23 (95% confidence interval 0.07-0.73). The two-year OS of the patients with a localized RT field was 63.6% and 9.1% for multiple RT fields (p= 0.00007). Four patients experienced grade ≥3 gastrointestinal toxicity related to re-irradiation (4/33=12.1%). We could not find any predisposing dosimetric value in the comparisons with and without toxicity.

Conclusions: The dose constraints presented in this study are relatively low rates of toxicity, which may be useful when planning re-irradiation. Especially, for the patients who could be treated with localized RT field, radical re-irradiation with a high curative dose is a good option. No dosimetric predisposing factor was found for radical re-irradiation of abdominopelvic lesions in the composite plan.

## Introduction

Abdominopelvic lymph node metastasis/recurrence occasionally occurs within the previously irradiated region during disease progression [1-3]. When other modalities, including surgical procedures and/or systemic therapy, fail to salvage the recurrent abdominopelvic lymph nodes, re-irradiation may be considered [1-3]. Re-irradiation was previously contraindicated because of a cumulated irradiated dose that may exceed the tolerance dose of organs at risk (OARs). However, with the advent of radiotherapy techniques, such as intensity-modulated radiation therapy (IMRT), it is possible to irradiate complex target organs with higher doses than with conventional radiation therapy (RT) [4-6]. Several reports have mentioned re-irradiation for tumors of the brain, spinal cord, and head and neck region [7]. However, few reports have been published on the re-irradiation of abdominopelvic lesions especially for lymph node recurrence [1-3,8-10].

Therefore, this study aimed to evaluate the outcomes and toxicity after re-irradiation for abdominal pelvic lymph node recurrence in patients who were not candidates for surgery or chemotherapy and to explore the effects of adverse events and dose factors using a combined duodenal and bowel dose-volume histogram (DVH).

## Materials and methods

Study design and patients

Between January 2008 and March 2017, 35 patients underwent re-irradiation of the abdominopelvic lymph node area with complete overlap of the previous RT in a single institution. Re-irradiation was performed in patients in whom lesions other than the target lesions for re-irradiation were controlled by chemotherapy. There was no concurrent use of anticancer drugs, including angiogenesis inhibitors, during re-irradiation. Of them, we excluded two patients whose initial RT records were not available for analysis. The remaining 33 patients were evaluated. Informed consent was obtained from all patients involved in the study at each institution. The study was conducted according to the guidelines of the Declaration of Helsinki and approved by the Institutional Review Board of the Kansai Medical University: IRB NO 2017140. Information on this study is publicly available on the facility's website.

The eligibility criteria of this retrospective study were as follows: (1) the patient had a recurrent tumor after definitive radiotherapy, (2) the tumor was unresectable (surgically impossible and/or patient's condition), and (3) the patient had no other active malignancy.

Radiation planning and therapy

Radiotherapy was performed using a 6-MV linear accelerator with fixed-field IMRT (Novalis BrainLAB AG, Feldkirchen, Germany). Patients underwent computed tomography (CT) scans and/or magnetic resonance imaging (MRI) before re-irradiation. Initial RT records in the Digital Imaging and Communication in Medicine (DICOM) format were obtained from the previous hospital. The small and large intestines were depicted with a loop approach on each of the 4D-CT at the time of simulation and the CT was taken on a different day to confirm the planning organ at risk volume (PRV) from the OAR. In the absence of DICOM records, the irradiation field was reproduced using Xio (Elekta AB, Stockholm, Sweden). The clinical target volume (CTV) was defined as the expansion of gross tumor volume (GTV) with a 0-0.5 cm margin considering the microscopic disease spread and the anatomical structure. In cases of multiple neighboring GTVs, the regional lymph nodes were enlarged as CTVs. The planning target volume (PTV) was defined as the CTV with a 0.3-0.6 cm margin.

A composite plan was prepared combining previous and current radiotherapy records superimposed in the current planning CT scan. The cumulative dose was recalculated. Additionally, we calculated a biologically equivalent dose of 2-Gy fractions (EQD2) using the following formula: EQD2 = D * (d + α/β)/(2.0 + α/β), where D is the total dose of a treatment course, d is the fraction dose, α/β = 3 Gy (Gy_3_) for OARs, and α/β = 10 Gy (Gy_10_) for the tumor. The OARs were also delineated using that CT scan. We performed a dosimetric analysis for the upper abdominal region adjacent to the duodenum for duodenal toxicity as well as an analysis for the intestine when the target was close to the large and small intestines. Re-irradiation planning protocol for target volume and OARs (duodenum, small and large intestines) was decided as follows: more than equal to 97% of the prescription dose was administered to the D95 (percentage of the minimum dose that covered 95% of the target volume) of PTV; minimal dose to the maximally irradiated doses delivered to 1 cc (D1 cc) and 5 cc (D5 cc) of OARs was set below 70 Gy_3_ and 50 Gy_3_, respectively; and D1 cc and D5 cc in the cumulative plans to OARs were 120 Gy_3_ and 100 Gy_3_.

**Figure 1:**
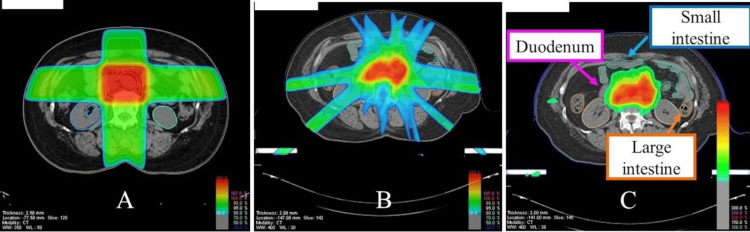
Typical scheme of re-irradiation (re-RT) planning (A) Previous radiotherapy planning record superimposed on the re-irradiation planning computed tomography (CT). (B) Re-irradiation radiotherapy record superimposed on the re-irradiation planning CT. (C) Cumulative dose displayed on the most recent planning CT. The organs at risk (duodenum, small and large intestines) were also delineated using that CT.

In the cumulative composite plan of the first and second RT, the following dosimetric data of the duodenum and intestines were extracted from the DVH: EQD2 60 Gy_3_, 66 Gy_3_, 72 Gy_3_, 78 Gy_3_, 84 Gy_3_, 90 Gy_3_, 100 Gy_3_, 110 Gy_3_, max dose, D1 cc and D5 cc. DVH of the duodenum was evaluated in 22 patients and that of the intestine in 32 patients (Table 1). The planning systems used were BrainSCAN™, iPlanRT Image ver.3.0.1 ™, iPlan RT Dose ver 4.1.2 ™ (BrainLab AG, Feldkirchen, Germany), and Xio. The cumulative radiation dose was summed using ShioRIS 2.0 TM (shioris.com, Japan) (Figure 1).

**Table 1:** Patient and treatment characteristics EQD2: a biologically equivalent schedule of 2-Gy fractions; GTV: gross tumor volumes; PTV: planning target volumes

	Total	Analyses for duodenal toxicity	Analyses for intestinal toxicity
Factor	N or Median	N or Median	N or Median
	n=33	n=22	n=32
Age	63 years (range: 32-84)	63 years (range: 32-84)	62 years (range: 32-84)
Sex			
Male	10 (30%)	7 (32%)	9 (28%)
Female	23 (70%)	15 (68%)	23 (72%)
Abdominal surgery			
+	23 (70%)	16 (73%)	22 (69%)
-	10 (30%)	6 (27%)	10 (31%)
Primary organ			
Cervical	18 (55%)	11 (50%)	18 (56%)
Colorectal	4 (12%)	2 (9%)	4 (13%)
Hepatocellular	3 (9%)	3 (14%)	2 (6%)
Bile duct	3 (9%)	2 (9%)	3 (9%)
Ovarian	2 (6%)	1 (5%)	2 (6%)
Pancreas	1 (3%)	1 (5%)	1 (3%)
Esophagus	1 (3%)	1 (5%)	1 (3%)
Lung	1 (3%)	1 (5%)	1 (3%)
Re-irradiated sites			
Para-aortic	21 (64%)	20 (91%)	20 (63%)
Iliac artery	9 (27%)	NA	9 (28%)
Obturator	1 (3%)	NA	1 (3%)
Mesenteric	1 (3%)	1 (5%)	1 (3%)
Hepatic hilum	1 (3%)	1 (5%)	1 (3%)
EQD2 prescription dose			
1st	58.4 Gy (range: 40.0-83.3)	58.0 Gy (range: 46.0-83.3)	57.2 Gy (range: 40.0-83.3)
2nd	70.4 Gy (range: 49.6-93.3)	70.4 Gy (range: 50.0-90.6)	70.4 Gy (range: 49.6-93.3)
Interval			
1st-2nd	15 months (range: 5-82)	12 months (range: 5-82)	14 months (range: 5-82)
GTV	49.7 cc (range: 2.1-390.2)	48.9 cc (range: 2.1-390.2)	49.9 cc (range: 2.1-390.2)
PTV	114 cc (range: 11-710)	117 cc (range: 11-710)	115 cc (range: 11-710)
The number of lesions			
Single	22 (67%)	15 (68%)	22 (69%)
Multiple	11 (33%)	7 (32%)	10 (31%)

Overall survival (OS) and toxicity assessment

Survival was measured from the date of the initial day of the second RT until the date of death or last follow-up if the patient was alive. Toxicity was scored with the Radiation Therapy Oncology Group (RTOG) toxicity criteria and the Common Terminology Criteria for Adverse Events (CTCAE), version 4.0. Toxicity of Grade 3 or higher was investigated. Toxicity was evaluated according to the following criteria: gastrointestinal (GI) bleeding, obstruction, perforation, and necrosis requiring hospitalization treatment; diarrhea; constipation; significant skin atrophy; capillary dilation; and ulceration.

Statistical analysis

Dates for continuous variables are shown as median (range). All statistical analyses were performed with EZR version 1.35 (Saitama Medical Center, Jichi Medical University, Saitama, Japan), which is a graphical user interface for R version 3.3.2 (The R Foundation for Statistical Computing, Vienna, Austria). If the lesions were irradiated within a single PTV region at a single isocenter, they were defined as localized RT fields, even if there were multiple GTVs. For example, if multiple lymph node metastases in the aortic lymph node region could be treated in a localized RT field, it was defined as localized. All other cases were defined as multiple. The relationships between the clinical or DVH parameters and toxicity were analyzed with the Mann-Whitney U test for quantitative variables and Fisher's exact test for categorical variables. The Kaplan-Meier method was used to analyze the OS rate using the log-rank test. Cox's proportional hazard model was used for the multivariate analysis. Factors with p-value < 0.05 in the univariate analysis were analyzed using the multivariate analysis. For all analyses, statistical significance was set at p < 0.05.

## Results

Patient characteristics

The patient and treatment characteristics are shown in Table 1. The median age was 63 years (range, 32-84 years). The major primary site of disease was cervical cancer (n = 18, 55%) and the major re-irradiation site was the para-aortic lymph nodes. The median dose of the previous RT was 58.4 Gy_10_(range: 40.0-83.3 Gy_10_). The median prescription dose of the second RT was 70.4 Gy_10 _(range: 49.6-93.3 Gy_10_) with a median interval of 15 months (range: 5-82 months) between the first and second RT. The median prescribed doses for localized and multiple groups were 71.5 Gy_10_ (range: 49.6-93.3 Gy_10_) and 65.0 Gy_10_ (52.0-74.4 Gy_10_). The median PTV was 114 cc (range: 11-710 cc). The median follow-up period from the second RT was 18 months (range: 2-156 months).

Toxicity

Four patients experienced grade ≥3 GI toxicity related to re-irradiation (4/33 = 12.1%), including one grade 5 duodenal ulcer and bleeding, two grade 3 duodenum obstruction, and one grade 3 rectal ulcer and radiation enteritis. No apparent tumor recurrence was observed in these four patients. In addition, four patients experienced grade ≥ 3 GI toxicities unrelated to re-irradiation (bleeding and perforation, and obstruction). There was no grade ≥ 3 skin toxicity. The results of the DVH parameter analysis of the duodenum and intestines are shown in Table 2. None of the DVH parameters was associated with grade 3 or higher toxicity.

**Table 2:** Comparison of dose-volume histogram (DVH) parameters of the duodenum and intestines in patients with and without toxicity. PTV: planning target volumes

	Duodenum								Intestine					
Toxicty	No (n=19)			Yes (n=3)					No (n=31)			Yes (n=1)		
	Median	Range		Median	Range		p.value		Median	Range				p.value
PTV	128.4 cc	26.3-710.2		114.4 cc	11.02-121.1		0.356		114.4 cc	11.0-710.2		136.8 cc		0.625
Dose														
Max	122.6 Gy_3_	56.2-190.8		115.2 Gy_3_	75.5-194.1		0.857		114 Gy_3_	57.7-188.8		137.9 Gy_3_		0.500
D1 cc	97.4 Gy_3_	41.5-179.7		80.6 Gy_3_	59.2-154.5		0.857		96.6 Gy_3_	43.4-181.0		135.4 Gy_3_		0.312
D5 cc	77.4 Gy_3_	22.5-158.8		63.7 Gy_3_	50.8-121.4		0.929		74.0 Gy_3_	25.7-169.1		130.4 Gy_3_		0.125
Volume														
V60 Gy_3_	13.1 cc	0-24.4		6.1 cc	0.8-22.6		0.924		13.6 cc	0-283.8		43.7 cc		0.812
V66 Gy_3_	8.1 cc	0-23.4		4.6 cc	0.2-19.6		0.773		7.7 cc	0-248.5		42.7 cc		0.448
V72 Gy_3_	6.3 cc	0-22.3		3.0 cc	0.0-16.8		0.923		5.5 cc	0-233.9		41.2 cc		0.386
V78 Gy_3_	4.9 cc	0-21.1		1.4 cc	0-14.4		0.735		4.3 cc	0-221.1		36.6 cc		0.327
V84 Gy_3_	3.9 cc	0-20.1		0.7 cc	0-12.1		0.735		3.2 cc	0-209.7		25.6 cc		0.276
V90 Gy_3_	2.9 cc	0-19.2		0.3 cc	0-10.4		0.771		2.6 cc	0-199.4		20.9 cc		0.230
V100 Gy_3_	0.7 cc	0-17.3		0.1 cc	0-8.3		0.883		0.7 cc	0-181.6		16.4 cc		0.154
V110 Gy_3_	0.2 cc	0-14.8		0.0 cc	0-6.7		0.883		0.1 cc	0-162.9		13.5 cc		0.113

Survival outcome

**Figure 2:**
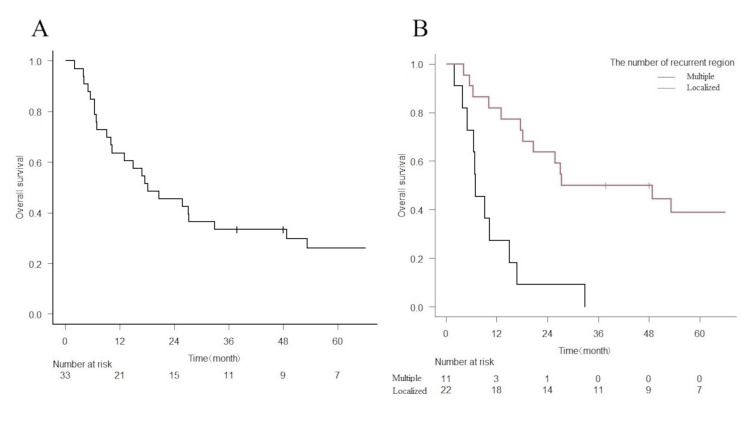
Overall survival rate after re-irradiation (A) Overall survival rate for all patients after re-irradiation of the abdominopelvic lesion. (B) Overall survival rates according to localized or multiple RT fields after re-irradiation (p = 0.00007).

The two-year OS rate was 45.5% (95% confidence interval (95% CI) = 28.2-61.2%) (Figure 2A). The univariate and multivariate analyses of various factors for OS rates are shown in Table 3. In the univariate analysis, significant differences were observed in the number of lesions (p = 0.00007) and PTV (p = 0.029). Additionally, the number of recurrent regions (localized or multiple, p = 0.006) remained significantly independent of the prognostic factors for OS rates in the multivariate analysis (Figure 2B). The two-year OS rates were 63.6% for localized RT fields (95% CI, 40.3-79.9%,) and 9.1% for multiple RT fields (95% CI) = 0.5-33.3%, p = 0.0000749, Figure 2B).

**Table 3:** Univariate and multivariate analyses of various factors for overall survival OS: overall survival; RT: radiation therapy; EQD2: biologically equivalent schedule of 2-Gy fractions; PTV: planning target volume; GTV: gross tumor volume

		Univariate analysis			Multivariate analysis	
	Number	Two-years OS				
Characteristics		rate (%)	P-value		HR (95% CI)	P-value
Age			0.347		NA	NA
≧60	18	56				
<60	15	33				
Sex			0.664		NA	NA
Male	10	50				
Female	23	44				
Abdominal surgery			0.801		NA	NA
+	23	39				
-	10	60				
Primary organ			0.626		NA	NA
Cervical	18	50				
Other	15	40				
Treatment site			0.210		NA	NA
Para-aortic	21	48				
Other	12	42				
The number of lesions			0.00007		0.23 (0.07-0.73)	0.013
Single	22	64				
Multiple	11	9				
Prescription dose (EQD2)			0.337		NA	NA
>65Gy	21	52				
<=65Gy	12	33				
PTV			0.029		1.23 (0.35-4.39)	0.749
<=110㏄	16	63				
>110㏄	17	29				
GTV			0.024		1.07 (0.28-4.18)	0.915
<=50㏄	17	65				
>50㏄	16	25				
Interval between 2nd RT			0.569		NA	NA
>12month	19	53				
<=12month	14	36				

## Discussion

In this retrospective study of 33 patients with abdominopelvic lymph node re-irradiation using IMRT, we found no predisposing dose constraint in the composite DVH for grade ≥3 toxicity for the duodenum and intestine. In addition, patients who could be treated with localized radiation treatment fields showed superior survival than those who required multiple RT fields. These findings may provide useful data on the safe and effective deliverance of high-dose re-irradiation using IMRT.

Several studies have reported on the tolerable dose to the OARs for initial radiotherapy. Lee et al. reported that the risk of duodenal perforation is increased if the D2 cc of the duodenum is greater than 60 Gy or if there is a duodenal ulcer prior to re-irradiation [11]. Verma et al. reported that dose-escalated IMRT can safely and effectively treat para-aortic nodal disease in gynecologic malignancies and limiting the volume of the duodenum receiving 55 Gy (V55) to below 15 cm^3^ may reduce the risk of duodenal complications [5].

However, little is known regarding the tolerable dose of the OARs in re-irradiation. Abusaris et al. proposed the following dose constraints in their two studies: the cumulative maximum dose of 90 Gy_3_ and 100 Gy_3_ for the bowel and rectum, respectively [1,2]. The University of Michigan had reported discounting OAR doses based on the interval of re-irradiation from the initial RT [10]; the discount factor was 10% and 25% after six months and one to three years from the initial RT for duodenal toxicity, respectively; 25% after six months from the initial RT in the small bowel toxicity; and 10%, 25%, and 50% after three months, six months, and one to three years, respectively, after the initial RT for rectal toxicity.

In this study, the dose constraint to the mucosal layer of the intestine was evaluated as D1 cc, and the dose constraint to the whole circumference of the intestine was evaluated as D5 cc. In addition, given that the initial RT prescribed around 50 Gy, which is a prophylactic dose, the re-irradiation dose constraints were defined by assuming that D1 cc could be irradiated up to nearly the radical dose and D5 cc up to the prophylactic dose. Therefore, we set the following dose constraints: D1cc and D5 cc for OARs were set below 70 Gy_3_ and 50 Gy_3_, respectively, and D1 cc and D5 cc for OARs in the cumulative plan were set as 120 Gy_3_ and 100 Gy_3_, respectively. We could speculate that our dose constraints were in line with those reported previously, because the median interval after the initial treatment was 15 months in our study.

In our study, 12.1% (4/33) of patients experienced grade ≥ 3 late toxicity related to re-irradiation; however, no correlation was found between the toxicity and dosimetric parameters in the composite plan. Therefore, our dose constraints were effective.

Most adverse events entailed toxicity related to the duodenum (Table 4). One reason may be that the duodenum is fixed to the retroperitoneum and is adjacent to the aorta, thus it may be susceptible to toxicity. The movable organs, such as the small and large intestines, may receive a lesser dose than the calculated dose. In the case of intestinal perforation, there is a high possibility that the pelvic area and intestinal tract are adhered to because of past surgery and RT. As we could not find any parameters related to grade ≥ 3 toxicity, re-irradiation was considered possible with few adverse events up to the tolerable doses of D1 cc and D5 cc used in this study. However, we suggest caution in the re-irradiation of recurrences near the duodenum or intestinal tracts with strong adhesions due to surgery.

**Table 4:** Characteristics of patients with toxicity RT: radiation therapy; fr: fraction; OAR: organ at risk

Sex	Treatment site	Toxicity	First RT	First fr	Second RT	Second fr	Interval between first and second RT (month)	Time to toxicity (month)	OAR dose associated with toxicity	
									D1 cc	D5 cc
Male	Para-aortic	Duodenum obstruction (Grade 3)	60	20	60	20	36	3	59.2	50.8
Female	Para-aortic	Duodenum obstruction (Grade 3)	60	20	60	20	6	3	80.6	63.7
Female	Iliac artery	Rectal ulcer and radiation enteritis (Grade 3)	50.4	28	60	20	25	7	135.4	130.4
Male	Hepatic hilum	Duodenal ulcer and bleeding (Grade 5)	50	10	50	5	8	6	154.5	121.4

Re-irradiation doses in the abdominopelvic region have been reported variably from 20 to 59.7 Gy, with many studies suggesting low prescribed doses [12-16]. This is probably because it was considered difficult to use high doses for re-irradiation, considering the tolerable doses of OARs. In cases of prescription doses of 60 Gy or more, improvement in OS is expected, necessitating treatment at higher doses [2,15]. The median dose in the current study was 70.4 Gy_10_, which is a relatively high dose, and OS was improved in the localized RT field patient group. We believe that patients with a localized RT field should be aggressively irradiated at high doses for radical re-irradiation. Shiba et al. reported 16 patients who received reirradiation by carbon-ion (C-ion) RT [3]. The median follow-up was 37 months and the median tumor size was 27 mm. No patient developed Grade 1 or higher acute toxicities and Grade 3 or higher late toxicities. The three-year OS, local control, and disease-free survival rates after C-ion RT were 74%, 94%, and 55%, respectively.

SABR-COMET trial had shown improved outcomes, including OS, using stereotactic body radiation therapy (SBRT) of oligometastatic disease (OMD) of various carcinomas [17]. OMD is generally defined as the presence of five or fewer transitions on an image [18]. However, clinical studies (NCT03721341, NCT04530513) are ongoing conducted to define OMD even when there are five or more metastases. Even multiple lesions may improve OS rates when controllable with re-RT. We hypothesized that this would be the case with re-RT. The advantage of SBRT is that small lesions can be treated with fewer fatal adverse events, on the other hand, IMRT may be advantageous for tumors at high risk of microscopic extension by allowing a larger treatment volume [19].

There are several limitations in this study. This was a retrospective study with small sample size, heterogeneity in terms of a wide variety of tumor subsites and histologies, and a limited follow-up time with different follow-up protocols. Because the assessment of adverse events was conducted by mail/telephone survey, only grade 3 or higher adverse events were included, and local control status was not confirmed in all cases. These factors may have prevented discrete conclusions and increased the risk of selection bias. Previous and current RT records were superimposed, and the OARs were delineated in the most recent planning CT scan. Therefore, we cannot exclude the possibility that the DVH parameters on treatment planning may have differed from the true doses. In the case of re-irradiation, two treatments are performed over a period of several months to several years, so a more complex position and dose analysis is required. In this paper, we use an offline IGRT method that analyzes the static DICOM-RT information of each irradiation with image-dose fusion software, but we hope that more dynamic online IGRT will be possible in the future if large amounts of data can be processed.

However, our study is one of the largest studies on re-irradiation for isolated abdominopelvic lymph node lesions; therefore, our study findings may be useful.

## Conclusions

The dose constraints presented in this study are relatively low rates of toxicity, which may be useful when planning high-dose re-irradiation. Especially, for the patients who could be treated with localized RT field, radical re-irradiation with a high curative dose is a good option. The present study has several limitations, including its retrospective study with the small number of patients analyzed. Accordingly, prospective studies are needed to determine long-term safety and efficacy for a larger number of homogeneous patient populations.
